# Memantine for Multiple Sclerosis: A Systematic Review and Meta-Analysis of Randomized Trials

**DOI:** 10.3389/fneur.2020.574748

**Published:** 2021-02-15

**Authors:** Christian Wilson R. Turalde, Adrian I. Espiritu, Veeda Michelle M. Anlacan

**Affiliations:** ^1^Department of Neurosciences, College of Medicine, Philippine General Hospital, University of the Philippines Manila, Manila, Philippines; ^2^Department of Clinical Epidemiology, College of Medicine, University of the Philippines Manila, Manila, Philippines; ^3^Department of Neurosciences, Center for Memory and Cognition, Philippine General Hospital, University of the Philippines Manila, Manila, Philippines

**Keywords:** memantine, multiple sclerosis, cognitive impairment, fatigue, spasticity

## Abstract

**Background:** Multiple sclerosis (MS), a disabling demyelinating disease of the central nervous system, is associated with cognitive impairment, spasticity, and fatigue. There are still no established guidelines on the management of MS-related sequela. Memantine has the potential to reduce glutamate toxicity, thereby reducing consequent cognitive impairment, spasticity, and fatigue.

**Objectives:** This study aims to determine the efficacy and safety of memantine in preventing cognitive impairment, reducing spasticity and fatigue, and controlling disability in MS patients through a review of relevant randomized trials.

**Methods:** MEDLINE, CENTRAL, Scopus, Embase, LILACS, ClinicalTrials.gov, and HERDIN were searched from inception to May 2020 for relevant trials.

**Results:** The search yielded 203 articles; four studies were included in the analysis. Pooled evidence shows that memantine compared with placebo does not significantly improve PASAT, ASS, MFIS, and EDSS scores of patients with MS. Memantine is associated with mild adverse drug events such as dizziness, fatigue, and anxiety.

**Conclusion:** There is not enough evidence to support the efficacy of memantine in preventing cognitive decline, controlling spasticity, reducing fatigue, and preventing disability. Future researches should consider the different MS subtypes, effect of co-administration of disease-modifying therapies, longer duration of administration, and more sensitive outcome measures to evaluate the potential benefit of memantine in MS.

## Introduction

Multiple sclerosis (MS) is a disabling inflammatory demyelinating disease affecting the central nervous system (CNS) (1). With an increasing prevalence of 50–300 per 100,000 individuals, it is the most common non-traumatic neurologic cause of permanent disability among young adults (2–4).

Cognitive impairment affects 45–65% of patients with MS causing considerable burden and disability (5). Previous studies suggest that although cognitive impairment is prevalent among patients with relapsing-remitting phenotype, more severe forms of impairment are observed among patients with primary progressive and secondary progressive diseases (6). It should be noted, however, that the widely used scale to assess disability in MS, the Expanded Disability Status Scale (EDSS), poorly describes cognitive impairment in MS (7). Early studies suggest that cognitive impairment in MS is a form of white matter subcortical dementia presenting with disruption in memory, processing speed, and executive function (8). A recent case–control study concluded that patients with MS have more pronounced deep gray matter atrophy compared with controls, supporting the claim that cognitive impairment in MS may also be due to cortical and deep gray matter lesions (9). Another proposed mechanism is glutamate excitotoxicity. This is supported by *in vivo* models which showed that a milieu of elevated glutamate activity causes neuronal excitotoxicity and subsequent cognitive decline (10).

There are still no established clinical guidelines on the management of cognitive impairment in MS. Recent studies suggest that disease-modifying therapies (DMT) significantly reduce the effect of neurodegeneration and subsequent cognitive impairment (11, 12). Studies investigating the role of drugs commonly used for dementia such as rivastigmine and donepezil for cognitive impairment in MS failed to demonstrate a significant benefit (13, 14).

Damage to the descending motor tracts brought about by autoimmune processes and subsequent neuroplasticity seen in MS is thought to cause disinhibition of motor reflexes leading to spasticity (15). Early studies involving rodent models of MS also suggest the role of glutamate toxicity in spasticity (16). Although cannabinoids, gamma amino butyric acid receptor agonists, imidazoline and alpha 2 receptor agonists, certain anti-epileptic drugs, and ryanodine receptor antagonists have been used for the symptomatic management of spasticity, there are still no specific guidelines on the treatment of MS-related spasticity (15).

Fatigue, or paucity of physical and mental drive, affects 83.1% of patients with MS (17). MS-related fatigue is understood to be related to axonal injury secondary to glutamate toxicity as manifested by the reduction in *N*-acetylaspartate levels seen in multivoxel spectroscopic studies (18, 19). Several non-pharmacologic modalities (cognitive behavioral therapy, exercise, and energy conservation) and pharmacologic agents (modafinil, amantadine, methylphenidate, and aspirin) have shown to decrease fatigue symptoms using several scales; however, the findings of these trials have been inconsistent (18).

Memantine, a non-competitive *N*-methyl-d-aspartate (NMDA) glutamate receptor antagonist, has been theorized to have a fundamental role in reducing glutamate toxicity (20). This agent has been the focus of recent clinical trials investigating its potential benefit in cognitive impairment (21, 22), spasticity (23), and fatigue (24) among patients with MS.

This study aims to determine the efficacy and safety of memantine among patients with multiple sclerosis in terms of improvement in cognitive function and reduction of spasticity, fatigue, and disability using a review of relevant trials.

## Methods

The PRISMA (Preferred Reporting Items for Systematic Reviews and Meta-Analyses) consensus guidelines were followed in this review (25).

### Criteria for the Selection of Studies for This Review

We considered clinical trials that employed randomized, double-blind, parallel group, placebo- and/or active-controlled designs in this review. Studies using other designs such as quasi-experimental, cluster-randomized, cross-over, prospective or retrospective cohort, case–control, and cross-sectional designs were excluded to prevent selection bias and the possibility of carryover effects of the intervention. We included trials involving patients who were diagnosed with multiple sclerosis satisfying the 2017 McDonald criteria (26, 27) regardless of the MS subtype (relapsing-remitting, secondary progressive, and primary progressive multiple sclerosis) including previous versions of this criteria. No restrictions in terms of age, sex, ethnicity, disease severity, and disease activity were employed. We included studies utilizing memantine *per orem* given at least 20 mg/day as the intervention compared with placebo and/or active agent/s. No restrictions in terms of concurrent or prior utilization of DMTs and other immunosuppressive drugs were implemented in this study. All trials tagged as primary researches, reported in English, and available as full-text articles were included.

### Outcome Measures Considered

- *Change in Paced Auditory Serial Addition Test (PASAT) score*—The PASAT is a neuropsychological test with a 0–60 scoring system with one-point increments. A positive change means improvement in information processing and sustained attention.
- *Change in Ashworth Spasticity Scale (ASS) score*—The ASS is a 0–4 scale with one-point increments for spasticity. A higher score signifies a higher degree of spasticity.
- *Change in Modified Fatigue Impact Scale (MFIS)* score—The MFIS is a 0–84 multidimensional scale used to assess perceived impact of fatigue in terms of physical, cognitive, and psychosocial aspects. A higher score means a higher perceived negative impact.
- *Change in Expanded Disability Status Scale (EDSS) score*—The EDSS is a 0–10 disability scale with 0.5-point increments wherein a higher score means greater degree of disability.
- *Adverse drug events (ADE)*—The proportion of participants who experienced any serious and non-serious adverse drug event after drug administration assessed at a defined time.

Other outcome measures that assess cognitive function and spasticity in patients with MS are summarized in the Supplementary Material.

### Search Methods for the Identification and Selection of Studies

The following electronic databases were searched for relevant trials: MEDLINE by PubMed, Cochrane Central Register for Controlled Trials (CENTRAL), Scopus, Embase, *Literatura Latino-Americana e do Caribe em Ciências da Saúde* (LILACS), ClinicalTrials.gov website, and HERDIN Database of the Philippines. The following general and MeSH term-based search strategy was employed: (memantine OR memantin OR 1,3-dimethyl-5-aminoadamantane OR 1-amino-3,5-dimethyladamantane OR namenda OR ebixa OR memantine hydrochloride OR axura OR D-145 OR D 145 OR D145) AND multiple sclerosis AND (randomized controlled trial OR control OR clinical trial OR random OR placebo OR trial OR groups OR assign OR allocation OR volunteer). Search strategies used in other databases are summarized in the Supplementary Material.

### Assessment of Risk of Bias, Data Collection, and Analysis

The Cochrane Collaboration Tool was used in the assessment of risk bias of the included studies.

The following details were collected and collated appropriately from the included trials: study design, participants, intervention details for the treatment group and the control/placebo group, and relevant outcomes described above.

Mean differences with 95% confidence intervals were used to measure treatment effect for the continuous outcomes, while risk ratios (RR) of benefit or harm with 95% confidence intervals were used for the dichotomous outcomes.

Syntheses of data were performed using the RevMan (computer program) (Version 5.4. Copenhagen: The Nordic Cochrane Center, The Cochrane Collaboration, 2014). Meta-analyses were performed using the fixed-effects model. For the continuous outcomes, the inverse variance method was used, while for the dichotomous outcomes, the Mantel–Haenszel method was employed. Statistical significance was reached if the 95% CI of the mean difference did not include the number zero for the continuous outcomes. For the dichotomous outcomes, statistical significance was noted if the 95% CI of the RR did not include the number one.

Clinical heterogeneity was assessed by comparing the population, intervention, comparison, and outcome measures in all the included studies. Methodological heterogeneity was evaluated by comparing the study designs and the risk of bias in the trials. Statistical heterogeneity was evaluated using the χ^2^-test with a *p*-value < 0.10 to indicate statistically significant heterogeneity. The degree of heterogeneity was measured using *I*^2^ statistic with values 0 to 40% indicating unimportant statistical heterogeneity (28).

## Results

### Included Studies

**Figure 1:**
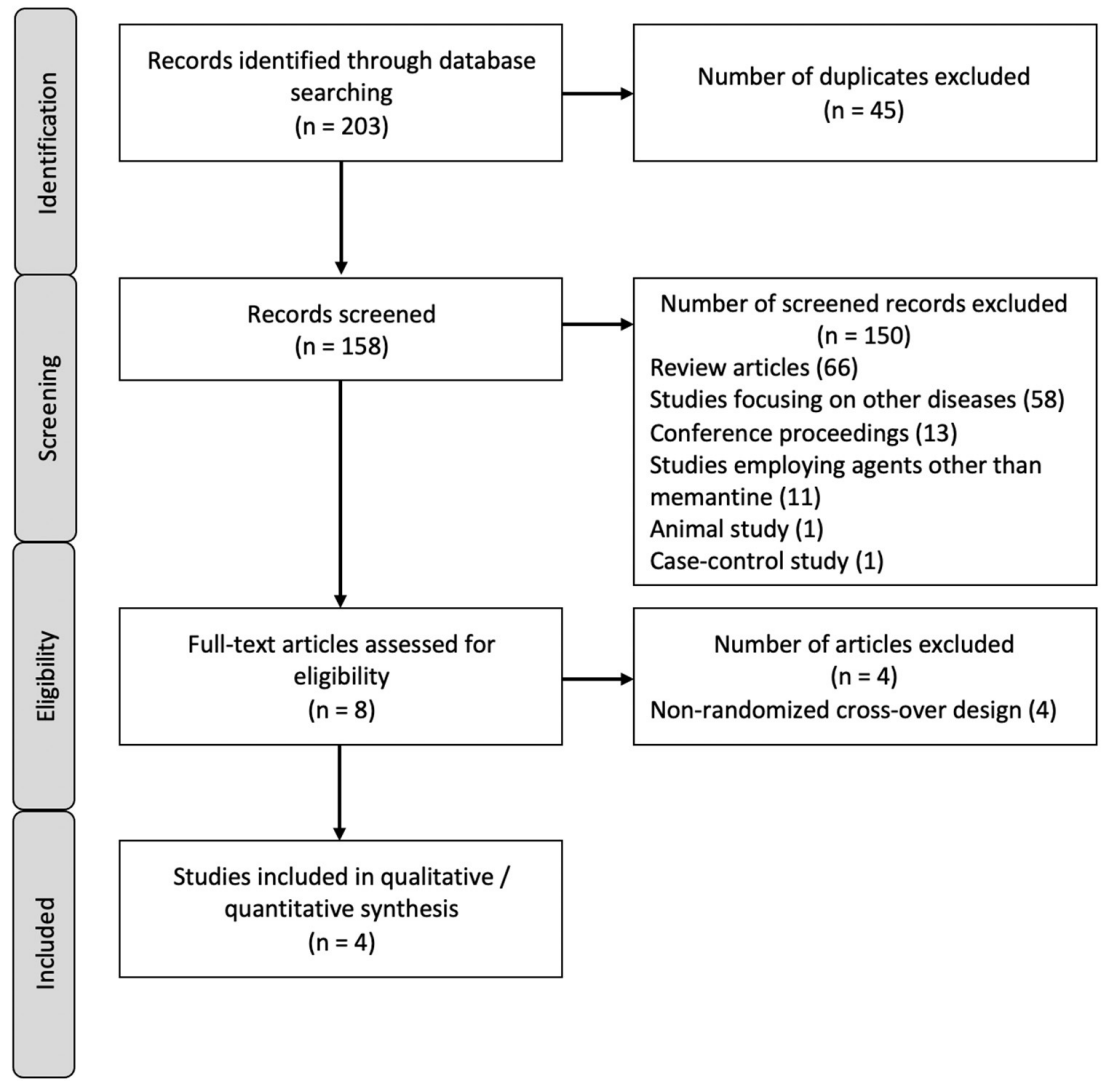
PRISMA flow diagram.

A total of 203 articles from major databases were retrieved. Forty-five records were identified as duplicates and were discarded. A total of 158 records were screened and 150 were excluded, of which 66 were review articles, 58 were studies that focused on diseases other than MS, 13 were conference proceedings, 11 were trials which studied drugs other than memantine, 1 was an animal study, and another was a case–control study. The remaining eight records were subjected to eligibility testing and four studies were excluded. A total of four studies were included in the qualitative and quantitative syntheses. The PRISMA flow diagram is shown in Figure 1. All the included studies employed a double-blind, randomized, placebo-controlled design (21–24). The characteristics of the included studies are summarized in Table 1, and the characteristics of the excluded studies are shown in the Supplementary Material.

**Table 1:** Included studies, corresponding treatment arms compared, sample characteristics, and outcome measures.

**First author/year**	**Treatment arms** **compared**	**Sample Size**	**Mean Age ±SD**	**F:M**	**Baseline EDSS**	**Primary Outcome**	**Secondary Outcomes**
Falsafi 2020	Memantine and placebo	32 (memantine), 32 (placebo)	35.9 ± 9.9 (memantine), 35.2 ± 7.6 (placebo)	5:1	1.0 (memantine), 1.0 (placebo)	MFIS score	ADE, BDI, EDSS, MSNQ
Saint Paul 2016	Memantine and placebo	48 (memantine), 38 (placebo)	39.6 ± 9.1 (memantine), 43.9 ± 7.9 (placebo)	2:1	3.1 (memantine), 3.4 (placebo)	PASAT score at 52 weeks	ADE, EDSS
Lovera 2010	Memantine and placebo	54 (memantine), 60 (placebo)	50.5 ± 8.2 (memantine), 50.4 ± 7.7 (placebo)	3:1	4.5 (memantine), 4.4 (placebo)	PASAT, CVLT-II, Victoria Stroop, SDMT, COWAT, and DKEFS scores	ADE
Mehta 2010	Memantine and placebo	11 (memantine), 10 (placebo)	52.9 ± 7.5 (memantine), 52.1 ± 12.2 (placebo)	1:1	5.5 (memantine), 5.3 (placebo)	ASS score after 12 weeks	PASAT

*ADE, adverse drug events; ASS, Ashworth spasticity scale; BDI, Beck depression inventory; CVLT-II, California verbal learning test-II; EDSS, expanded disability status scale; F:M, female to male ratio; LDFR, long delay free recall; MSNQ, multiple sclerosis neuropsychological questionnaire; PASAT, paced auditory serial addition test*.

### Population Characteristics in the Included Studies

A total of 285 patients were analyzed in the four trials. Table 2 summarizes the characteristics of the patients in the included trials. The majority were females with relapsing-remitting MS subtype. North Americans and Europeans comprise the majority of the participants. The baseline mean EDSS score ranged from 1.0 to 5.5.

**Table 2:** Characteristics of patients in the included studies (*N* = 285).

**Characteristics**	**Memantine (%)**	**Placebo (%)**
Sample (*n*)	145 (100)	140 (100)
**Age, years**		
Mean	43.85	45.28
**Sex**		
Female	112 (77)	104 (74)
Male	33 (23)	36 (26)
**Disease duration, years**		
Mean^*^	11.47	10.83
**MS subtype**		
RR	108 (74)	117 (84)
PP	12 (8)	6 (4)
SP	14 (10)	7 (5)
Data not available	11 (8)	10 (7)
**DMT**		
Interferon beta 1-a	8 (5.5)	11 (7.9)
Glatiramer	4 (2.8)	3 (2.1)
Dimethyl fumarate	1 (0.7)	2 (1.4)
Fingolimod	11 (7.6)	4 (2.9)
None	8 (5.5)	12 (8.6)
Data not available	113 (77.9)	108 (77.1)

* *Data on disease duration are extracted from 178 patients only (86 from the Memantine group and 92 from the placebo group) as only two trials specified disease duration as variables. DMT, disease-modifying therapies; MS, multiple sclerosis; PP, primary progressive; RR, relapsing-remitting; SP, secondary progressive*.

### Interventions Employed in the Included Studies

All studies compared memantine and placebo. Three trials used the same memantine titration schedule: 5 mg once daily during the first week, 5 mg twice daily during the second week, 5 mg in the morning and 10 mg in the evening during the third week, and 10 mg twice daily thereafter. One trial administered memantine at 10 mg daily for the first week followed by 20 mg daily throughout the duration of the trial (24). One trial allowed dose reduction based on the maximum tolerated dose (21). One trial administered memantine for 52 weeks (22), while the rest administered the treatment for 12 weeks.

### Assessment of Risk of Bias

**Figure 2:**
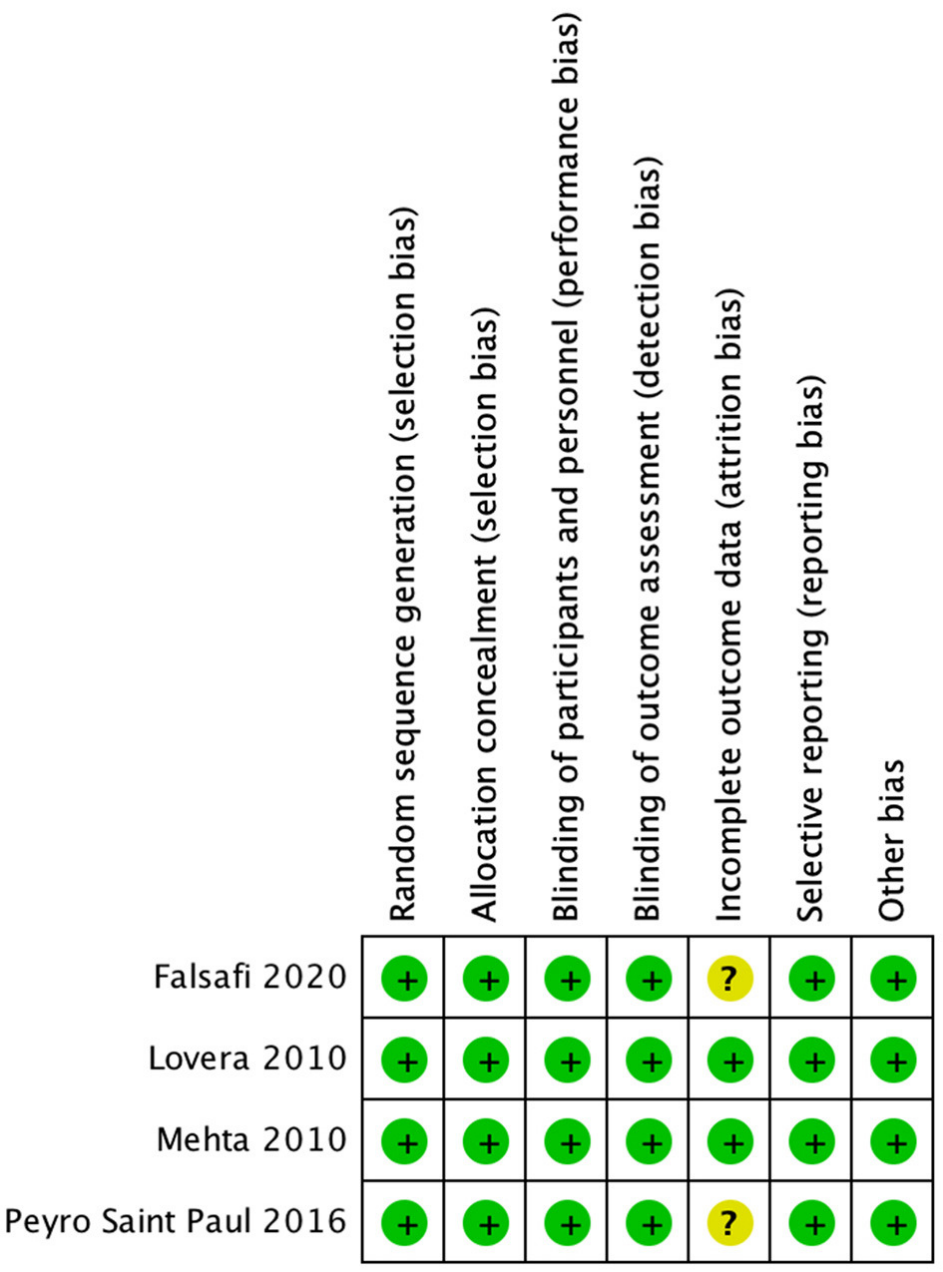
Risk of bias summary.

All four trials are deemed to have low risk for selection, performance, and detection biases as all of them employed a randomized double-blind design. Two studies are deemed to have unclear risk for attrition bias given the attrition rates of 28 and 38% in the placebo and treatment groups, respectively, in the study by Saint Paul et al. (22) and 25 and 50% in the placebo and treatment groups, respectively, in the study by Falsafi et al. (24). No other potential risk of bias is noted in the included trials. Figure 2 summarizes the risk of bias assessment.

### Effects of the Intervention

#### Effectiveness of Memantine in Preventing Cognitive Impairment Among Adult Patients With MS

**Figure 3:**
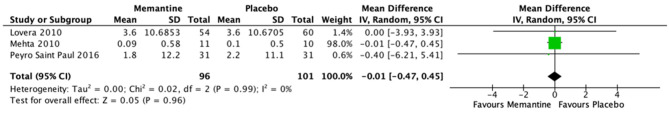
Forest plot of mean difference in PASAT scores for studies comparing memantine and placebo.

Three studies were merged to evaluate the effect of memantine compared with placebo on cognitive function of MS patients using the PASAT score. No significant difference in mean difference of PASAT score is noted between the memantine and placebo groups [MD (95% CI) = −0.01 (−0.47, 0.45), *p* = 0.99, *I*^2^ = 0%] (see Figure 3). One study (21) utilized other outcome measures for cognitive functions: California Verbal Learning Test–II (CVLT-II), Victoria Stroop, Symbol Digit Modalities Test (SDMT), Controlled Oral Word Association Test (COWAT), and Delis–Kaplan Executive Function System (DKEFS). There were no significant differences in mean changes of scores between the memantine and placebo groups [difference −0.6 (95% CI −2, 0.8), 0.0 (−0.2, 0.2), −0.4 (−3.2, 2.3), 0.4 (−3.5, 4.3), and −0.3 (−0.9, 0.3), respectively]. The trial by Falsafi et al. (24) also did not find a significant difference in mean change of MSNQ score between the memantine and placebo groups [difference −4.3 (95% CI −10.0 to 1.5)].

#### Effectiveness of Memantine in Reducing Spasticity Among Adult Patients With MS

Of the four included studies, only the trial by Mehta et al. (23) assessed the effect of memantine on spasticity. The mean difference in ASS scores between the placebo (1.0, SD 2.67) and memantine (1.55, SD 2.81) groups before and after 12 weeks of treatment is not significantly different (*p* = 0.65; 95% CI −1.96, 3.05).

#### Effectiveness of Memantine in Reducing Fatigue Among Adult Patients With MS

One trial studied the effect of memantine in MS-associated fatigue. Mean change from baseline MFIS score is not significantly different between the treatment and control groups [between-group difference = −1.9, 95% CI (−11.7 to 7.8), *p* = 0.702].

#### Effectiveness of Memantine in Reducing Disability Among Adult Patients With MS

In the two trials that evaluated the effect of memantine on disability using EDSS, no significant difference is observed in the mean difference of the baseline and post-treatment EDSS scores between the memantine and placebo groups [adjusted mean difference = −0.28, 95% CI (−0.72, 0.17), *p* = 0.22, *N* = 86] (22). In another trial, there was also no significant difference in the mean difference of the baseline and post-treatment degree of disability between groups using the MSFC outcome measure [mean difference = −0.06, 95% CI (−0.27, 0.16), *p* = 0.58, *N* = 21] (23).

#### Safety of Memantine Among Adult Patients With MS

**Figure 4:**
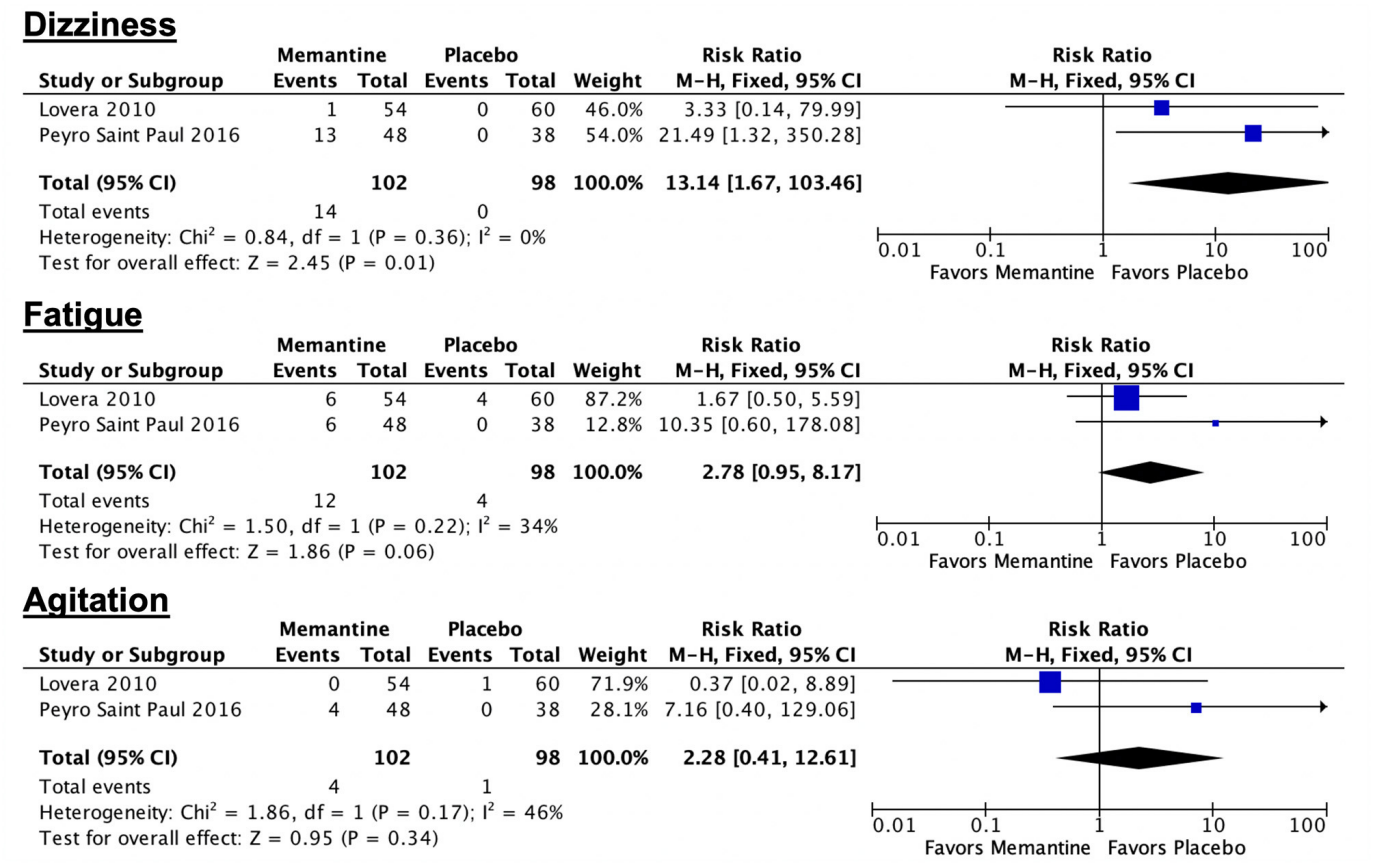
Forest plot summarizing combinable adverse drug effects from two trials.

The rates of adverse drug events among patients allocated to memantine range from 0 to 27.1% (21, 22). The most common ADEs are as follows: dizziness (1.85–27.1%), headache (27.1%), bladder infection (12.96%), fatigue (11.11–12.5%), diarrhea (10.4%), cough (9.26%), agitation (8.33%), somnolence (5.56%), ataxia (5.56%), anxiety (4.17%), constipation (3.7%), spasticity (3.7%), and rash (3.7%). The rates of other rare ADEs, including speech impairment, confusion, dizziness, and nervousness, range from 1.85 to 2.08%. In one trial involving the administration of memantine for 12 weeks, the rate of ADEs in the memantine group is not significantly different from that of the control group (21). In a study involving the administration of memantine for 52 weeks, the rates of the following ADEs in the memantine group are significantly higher compared with the rates in the placebo group: dizziness (*p* = 0.0005), headache (*p* = 0.023), and fatigue (*p* = 0.032) (22). Forest plots of combinable data comparing memantine and placebo in terms of adverse drug events are shown in Figure 4. Pooled evidence indicates that the number of patients who experienced dizziness is significantly higher in the memantine group compared with that in the placebo group (*p* = 0.01; 95% CI 1.67, 103.46). The rates of fatigue and agitation in the memantine group are higher than those in the placebo group, although the differences are not statistically significant (*p* = 0.06; 95% CI 0.95, 8.17 and *p* = 0.34; 95% CI 0.41, 12.61, respectively). In terms of serious adverse events, one episode of seizure was noted in a patient in the placebo group in one trial (22). No deaths occurred in the included trials.

## Discussion

This review provides comprehensive evidence from pooled results of four studies on the efficacy and safety of memantine in preventing cognitive decline, reducing spasticity and fatigue, and controlling disability among adult patients with multiple sclerosis.

The pathophysiology that led to the consideration of memantine, an NMDA receptor antagonist that blocks glutamate in neurons, as a potential agent for the prevention of cognitive impairment in patients with MS arose from the hypothesis that states that cognitive impairment in MS may be related to the excessive glutamate noise in the CNS plaques (29). Recent studies suggest that disease-modifying therapies significantly reduce the effect of neurodegeneration and subsequent cognitive impairment in MS. The BENEFIT (11), AFFIRM, and STRATA (12) trials emphasize the positive effect on cognitive functioning in the long-term follow-up of MS patients treated with interferon B-1b and natalizumab. Studies investigating drugs commonly used for dementia, however, failed to demonstrate a significant benefit for cognition (13, 14). There is not enough evidence to support the role of memantine in improving cognitive function of patients with MS as measured by mean difference in PASAT scores and other measures: CVLT-II, Victoria Stroop, SDMT, COWAT, and DKEFS. One possible reason is the timing of memantine administration. Animal studies involving encephalomyelitis rodent models suggest that the protective effect of NMDA blockade for cognitive deterioration in the setting of neuroinflammatory diseases is evident if carried out in the initial stages of neuroinflammation (30). The trials failed to segregate patients into those with early disease and with relapsing or progressive course. Another possible reason why the potential benefit of memantine is not demonstrated in the pooled evidence is the lack of subpopulation analysis in terms of DMT utilization. Inflammatory mediators such as tumor necrosis factor stimulate glutamate secretion and excitotoxicity (31). Glutamate blockade alone may not be enough in preventing cognitive deterioration. This is supported by studies demonstrating the protective effects of ocrelizumab (32), alemtuzumab (33), interferon B-1b (11), and natalizumab (12) on cognition of MS patients on long-term follow-up. The authors postulate that a subgroup of patients might benefit from memantine in terms of protection against cognitive deterioration—patients with early disease and receiving DMTs. To date, there are still no studies in the literature that define early MS disease. Future studies may look into the role of 7-T magnetic resonance imaging for early diagnosis of MS (34).

It is important to note that DMT initiation can be associated with paradoxical brain pseudo-atrophy which is understood to be due to fluid shifts and resolution of edema as neuroinflammation subsides (35). This is different from true brain atrophy which is a true marker of extensive demyelination, axonal loss, and degeneration (36). True brain atrophy, specifically thinning of the fronto-parietal cortical regions, precuneus atrophy (37), and thalamic atrophy (38) are significantly correlated with cognitive impairment. To date, evidence on the difference in outcomes of memantine treatment in patients with pseudo-atrophy compared with those with true brain atrophy is still lacking. It is logical to postulate, however, that better cognitive outcomes may be expected if memantine treatment is initiated early on than if started when true atrophy from neurodegeneration is already apparent.

Another possible reason for the non-demonstration of clinical benefit is the relatively short duration of treatment. Among patients with Alzheimer's disease dementia, improvement in cognitive function is already evident as early as 12 weeks of administration of memantine (39). This scenario may not be applicable among patients with MS as their decline in cognitive functions is tethered to the number of relapses or attacks and is understood to progress more slowly compared with the steady gradual decline seen among patients with Alzheimer's disease dementia. The period of 12 to 52 weeks of memantine administration and observation may not be enough. The severity of cognitive impairment may also be very different as patients with MS may present with mild to moderate impairment in contrast to patients with Alzheimer's disease dementia who usually present with mild to severe impairment. With a sensitivity of 74% in detecting cognitive impairment (40), the PASAT measure may not be perfectly perceptive in detecting subtle changes in cognitive functioning in the mild to moderate arena of the cognitive functioning spectrum. Lastly, another possible reason can be due to non-concordance of the pathophysiology. Although high levels of glutamate noise—the target of memantine—are seen in spectroscopic studies of white matter lesions in MS, this may not be the only contributing process to cognitive impairment. Recent studies suggest other contributory pathophysiologic features such as thalamic degeneration, hippocampal changes, synaptic loss (8, 41), and reduced GABA levels (31), hence the ineffectiveness of memantine in improving cognitive function or in preventing cognitive impairment.

This review demonstrates that short-term memantine administration does not significantly reduce spasticity among MS patients. One explanation is the possibility of non-concordance in the pathophysiology. Although studies involving animal models of MS demonstrate that antagonism in the NMDA receptor reduces muscle tone (16, 42), more complex pathophysiologic processes such as dynamic changes in the levels of cytokines, prostaglandins, and reactive oxygen species have been implicated in the pathophysiology of spasticity in MS (15). Moreover, with a sensitivity in detecting spasticity of just 50% (43), the zero- to four-point ADSS may not be able to detect subtle changes in spasticity.

Pooled evidence failed to show that memantine can reduce the degree of MS-related fatigue as measured by MFIS. One reason is that fatigue is theorized to be tethered to depression and cognitive symptoms as presented in the model by Brenner and Piehl (44). Treating fatigue as just a consequence of excessive glutamate noise in MS with memantine may not be sufficient. Another reason again may be the possibility of non-concordance in pathophysiology as MS-related fatigue is not only correlated with glutamate toxicity but also with gray and white matter atrophy (18). It is important to note that memantine may actually exacerbate the symptoms of fatigue. Two of the trials in this review showed that the rate of fatigue among patients in the memantine group is higher than that in the placebo group, though this is not statistically significant (21, 22).

In terms of disability, evidence in this review failed to show benefit from short-term administration of memantine (12–52 weeks) compared with placebo. This may be due to the recruited participants. In the trial which evaluated pre- and post-treatment EDSS, the baseline level of disability is within the mild spectrum with mean EDSS score ranging from 3.1 to 3.2 (22). Furthermore, all recruited participants in the said study belong to the relapsing-remitting clinical subtype, a subgroup that generally has mild forms of disability.

The role of memantine in MS according to the 2014 National Institute of Health Care and Excellence clinical guideline on the management of MS in adults (CG186) is limited to being a second-line treatment of MS-related nystagmus and oscillopsia, second to gabapentin (45). This recommendation is backed by studies on acquired and congenital nystagmus in general using retrospective and non-randomized cross-over designs (46–48). At the time of writing, there is insufficient evidence to support the beneficial role of memantine in MS-related cognitive impairment, spasticity, fatigue, and disability.

Pooled evidence in this study shows that memantine is associated with non-serious adverse drug events such as dizziness, fatigue, and agitation. This is consistent with the findings in other studies on memantine in multiple sclerosis. In a pilot study by Villoslada and collegues (49) involving 19 patients with MS, memantine at a higher dose of 30 mg/day was associated with blurred vision, fatigue, severe headache, increased muscle weakness, walking difficulties, or unstable gait. These events led to the pre-mature termination of the trial. Conceivably, the high rate of adverse events in the pilot study is due to the quick titration of memantine, with incremental increase of 10 mg per day after 1 week. This review provides evidence demonstrating that memantine administration is not associated with any serious adverse drug events or death when administered following proper titration.

## Conclusion

There is not enough evidence to support the role of memantine at a dose of 20 mg per day administered for 12–52 weeks among patients with MS in preventing cognitive deterioration, controlling spasticity, reducing fatigue, and improving the degree of functionality compared with placebo. Memantine administration, though associated with minor adverse drug events such as dizziness, fatigue, and anxiety, is generally safe among patients with MS.

Further researches investigating the different MS clinical subtypes, role of co-administration of memantine with DMTs, longer duration of administration, and more unified and sensitive outcome measures are needed to evaluate the potential benefit of memantine among patients with MS.

## Data Availability Statement

The original contributions generated in the study are included in the article/Supplementary Material, further inquiries can be directed to the corresponding author/s.

## Author Contributions

All authors have been sufficiently involved in this work to take responsibility for its validity and final presentation as an original publication.

## Conflict of Interest

The authors declare that the research was conducted in the absence of any commercial or financial relationships that could be construed as a potential conflict of interest.

## Abbreviations

ADE: adverse drug events
AFFIRM: Natalizumab Safety and Efficacy in Relapsing-Remitting Multiple Sclerosis
ASS: Ashworth Spasticity Scale
BENEFIT: Betaseron/Betaferon in Newly Emerging MS For Initial Treatment
BDI: Beck Depression Inventory
CENTRAL: Cochrane Controlled Register of Trials
COWAT: Controlled Oral Word Association Test
CVLT-II: California Verbal Learning Test–II
DKEFS: Delis–Kaplan Executive Function System
DMT: disease-modifying therapies
EDSS: Expanded Disability Status Scale
HERDIN: Health Research and Development Information Network
LDFR: long delay free recall
LILACS: *Literatura Latino-Americana e do Caribe em Ciências da Saúde*
MFIS: Modified Fatigue Impact Scale
MS: multiple sclerosis
MSFC: Multiple Sclerosis Functional Composite
MSNQ: Multiple Sclerosis Neuropsychological Questionnaire
PASAT: Paced Auditory Serial Addition Test
PRISMA: Preferred Reporting Items for Systematic Reviews and Meta-Analyses
SDMT: Symbol Digit Modalities Test
STRATA: Safety of TYSABRI Re-dosing and Treatment.
