# Supplementary material for: Memantine for Multiple Sclerosis: A Systematic Review and Meta-Analysis of Randomized Trials
Source: Front Neurol. 2021 Feb 15;11:574748. doi: 10.3389/fneur.2020.574748 (PMC7917060; doi:10.3389/fneur.2020.574748)
Supplement: Supplementary file 1 [file Data_Sheet_1.docx]

**Supplementary Material A**

**SEARCH STRATEGY**

**Cochrane Central Register of Controlled Trials (CENTRAL) (by The Cochrane Library)**

#1 MeSH descriptor: [Memantine] explode all trees (342)

#2 MeSH descriptor: [Multiple Sclerosis] explode all trees (2996)

#3 #1 AND #2 (6)

**MEDLINE (by PubMed)**

#1: memantine OR Memantin OR 1,3-Dimethyl-5-aminoadamantane OR 1-Amino-3,5-dimethyladamantane OR Namenda OR Ebixa OR “Memantine Hydrochloride” OR Axura OR D-145 OR “D 145” OR D145

#2: “Multiple Sclerosis, Chronic Progressive” OR “Multiple Sclerosis, Relapsing-Remitting”

#3 randomi?ed control* trial* OR control* clinical trial* OR clinical trial* OR random* OR placebo* OR trial* OR groups OR assign* OR allocat* OR volunteer*

#4 #1 AND #2 AND #3

**Scopus**

#1: memantine OR Memantin OR 1,3-Dimethyl-5-aminoadamantane OR 1-Amino-3,5-dimethyladamantane OR Namenda OR Ebixa OR “Memantine Hydrochloride” OR Axura OR D-145 OR “D 145” OR D145

#2: “Multiple Sclerosis, Chronic Progressive” OR “Multiple Sclerosis, Relapsing-Remitting”

#3 randomi?ed control* trial* OR control* clinical trial* OR clinical trial* OR random* OR placebo* OR trial* OR groups OR assign* OR allocat* OR volunteer*

#4 #1 AND #2 AND #3

**EMBASE**

(memantine OR memantin OR 1,3-dimethyl-5-aminoadamantane OR 1-amino-3,5-dimethyladamantane OR namenda OR ebixa OR memantine hydrochloride OR axura OR D-145 OR D 145 OR D145) AND multiple sclerosis AND (randomized controlled trial OR control OR clinical trial OR random OR placebo OR trial OR groups OR assign OR allocation OR volunteer)

**LILACS (*Literatura Latino-Americana e do Caribe em Ciências da Saúde*) (IAH format)**

1 memantine

2 “multiple sclerosis”

3 ((pt randomized controlled trial OR pt controlled clinical trial OR pt multicenter study OR mh randomized controlled trials as topic OR [mh controlled clinical trials as topic] OR [mh multicenter study as topic] OR [mh random allocation] OR [mh double-blind method]) OR ((ensaio$ OR ensayo$ OR trial$) AND (azar OR acaso OR placebo OR control$ OR aleat$ OR random$ OR enmascarado$ OR ((duplo$ OR doble$ OR double$) AND (cego OR ciego OR blind OR mask))) AND clinic$))

4 1 AND 2 AND 3

**ClinicalTrials.gov U.S. National Library of Medicine**

Memantine | Interventional Studies | Multiple Sclerosis

**Supplementary Material B**

**OTHER OUTCOME MEASURES CONSIDERED**

| **Outcome Measure** | **Aspect measured** | **Comments** |
| --- | --- | --- |
| **CVTL-II** (California Verbal Learning Test–II) | A test for verbal memory | This test has high sensitivity but is a poor candidate for future unsupervised assessment (29). |
| **Victoria Stroop** | A test for executive function | This test is considered appropriate for geriatric population and for patients with dementia who are prone to exhaustion during neuropsychological tests (38). |
| **SDMT** (Symbol Digit Modalities Test) | A test of cognitive processing speed | A clinically meaningful change is seen in score difference of 3. This test has a very high sensitivity, good to excellent reliability; hence, is recommended monitoring tool in clinical practice (29). |
| **COWAT** (Controlled Oral Word Association Test) | A test for verbal fluency | This test has moderate sensitivity. It is not recommended for screening or monitoring in clinical practice (29). |
| **DKEFS** (Delis-Kaplan Executive Function System) | A test for executive function | This test has moderate sensitivity. It is not recommended for brief monitoring in clinical practice and in clinical trials (29) |
| **MSFC** (Multiple Sclerosis Functional Composite) | A composite measure combining scores for leg function/ambulation, arm/hand function, and cognitive function. | This test is considered a valid measure of level of disability in patients with MS. |
| **MSNQ** (Multiple Sclerosis Neuropsychological Questionnaire) | A self-report questionnaire on behavioral and cognitive problems | The cut-off scores are influenced by the educational attainment of the patients (39). |

**Supplementary Material C**

**CHARACTERISTICS OF EXCLUDED STUDIES**

| **Study** | **Methods** | **Participants** | **Interventions** | **Outcome Measures** | **Reason for exclusion** |
| --- | --- | --- | --- | --- | --- |
| **Villoslada 2009**  Memantine induces reversible neurologic impairment in patients with MS | Pilot trial, supposedly randomized, double-blind, cross-over study | 19 diagnosed cases of multiple sclerosis | Memantine 30 mg versus Placebo | Supposedly Brief Repeatable Battery–Neuropsychology | Premature termination; cross-over design |
| **Tilikete 2012**  Treatment of Pendular Nystagmus With Gabapentin and Memantine in Patients With Multiple Sclerosis | Open label, randomized, cross-over study | 10 diagnosed cases of multiple sclerosis | Memantine versus Gabapentin with washout period of 8-11 days | Velocity of nystagmus using eye movement recording | Open label, cross over design |
| **Starck 2009**  Acquired pendular nystagmus in multiple sclerosis: an examiner-blind cross-over treatment study  of memantine and gabapentin | Prospective examiner-blind, cross-over study | 11 diagnosed cases of multiple sclerosis  (F=7, M=4) Mean age is 44.9  Mean EDSS is 6.1 | Memantine 10 mg versus Gabapentin 200 mg  Cross-over design with 5-day washout period | Amplitude and frequency of acquired fixational pendular nystagmus as measured by electrooculography | Cross-over design |
| **Starck 1997**  Drug therapy  for acquired pendular nystagmus in multiple sclerosis | Non-randomized observational study | 14 diagnosed cases of multiple sclerosis  (F=11, M=3)  Mean age is 43.1  Mean EDSS is 5.8 | Scopolamine plaster per right mastoid for 3 days versus Memantine 15-60 mg PO for 7 days | Amplitude and frequency of acquired fixational pendular nystagmus as measured by electrooculography | Non-randomized observational design |
